# Supplementary material for: Enhanced Bandwidth Broadening of Infrared Reflector Based on Polymer Stabilized Cholesteric Liquid Crystals with Poly(N-vinylcarbazole) Used as Alignment Layer
Source: Polymers (Basel). 2021 Jul 8;13(14):2238. doi: 10.3390/polym13142238 (PMC8309408; doi:10.3390/polym13142238)
Supplement: Supplementary file 1 [file polymers-13-02238-s001.zip › polymers-1283857-SI.pdf]

Support Information

For

Enhanced Bandwidth Broadening of Infrared Reflector Based on polymer Stabilized Cholesteric liquid Crystals with Poly(N-vinylcarbazole) Used as Alignment Layer

Limin Zhang [1,2], Qiumei Nie [1], Xiaofang Jiang [3], Wei Zhao [1,2], Xiaowen Hu [1,2,*], Lingling Shui [2], Guofu Zhou [1,2]

1 SCNU-TUE Joint Lab of Device Integrated Responsive Materials (DIRM), National Center for International Research on Green Optoelectronics, South China Normal University, Guangzhou 510006, China

2 Guangdong Provincial Key Laboratory of Optical Information Materials and Technology & Institute of Electronic Paper Displays, South China Academy of Advanced Optoelectronics, South China Normal University, Guangzhou 510006, China

3 Laboratory of Quantum Engineering and Quantum Material, School of Physics and Telecommunication Engineering, South China Normal University, Guangzhou 510006, China

* Correspondence: Xiaowen Hu, xwhu@m.scnu.edu.cn

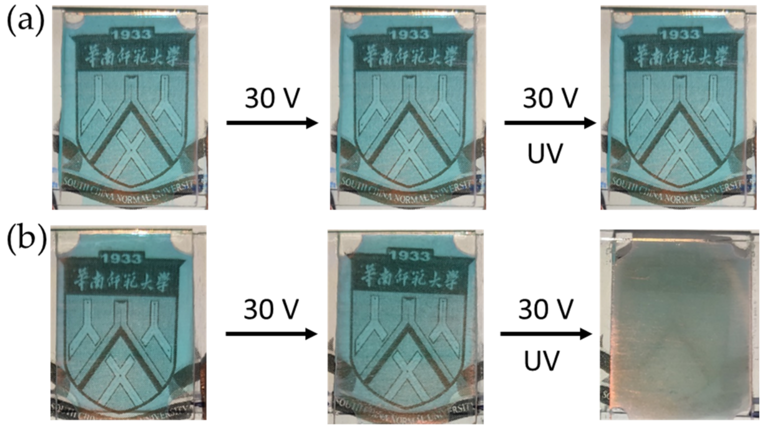

Fig. S1 For both PVA–PVA-based CLC cell and PVA–PVK-based CLC cell, at applied voltage of 30V, both cells stay in colored and transparent state due to cholesteric texture. When shining UV light with intensity of 10 mW/cm^2 for 60s: (a) the PVA–PVA-based CLC keeps initial state; (b) the PVA–PVK-based CLC cell turns opaque due to focal conic state.

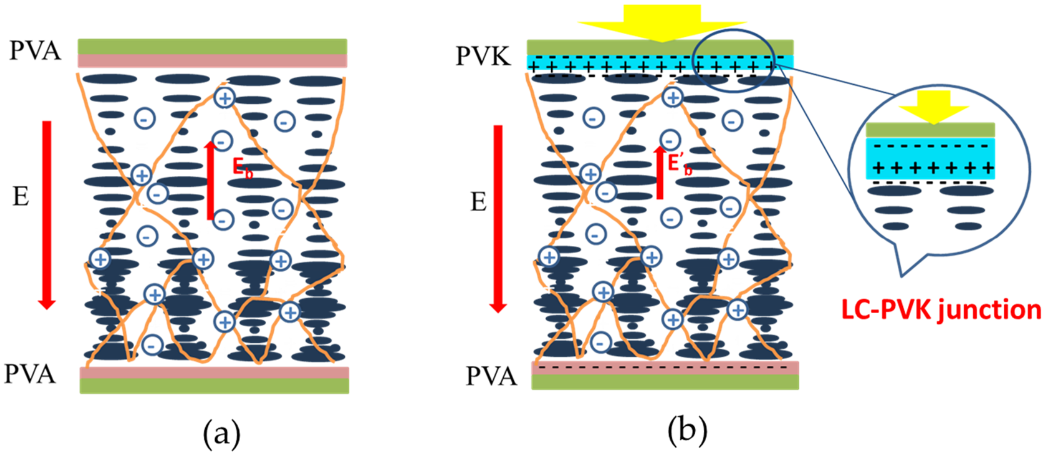

Fig. S2 In the PVA–PVK cell (b), the generated hole–electron pairs in the PVK layer in the presence of UV light can neutralize the impurity electrons in the LC–PVK junction; therefore, the built-in electric field E_b'in the PVA–PVK cell is smaller than that (E_b) in PVA–PVA cell (a). On application of the same electric field, the effective electric field (E_{eff}) in the PVA–PVK cell is larger than that in PVA–PVA cell, which results in enhanced bandwidth broadening.

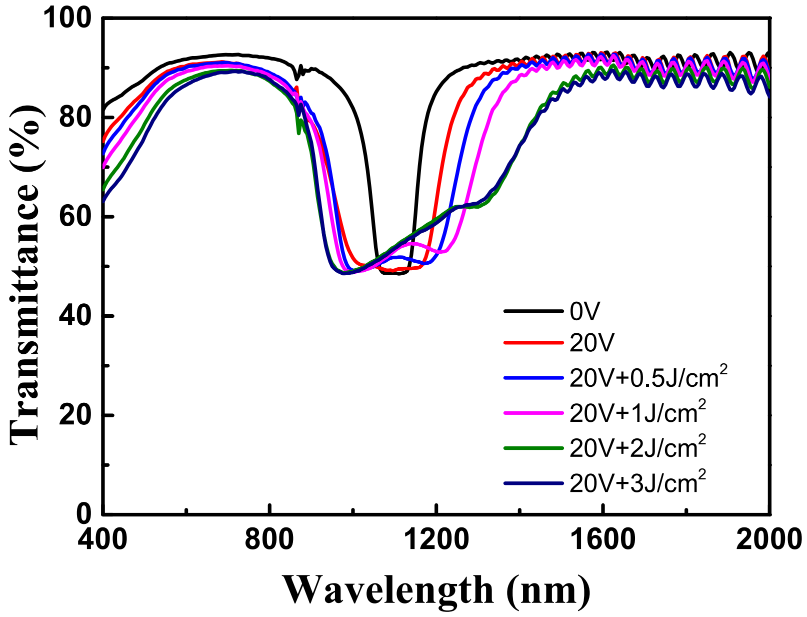

Fig.S3 The transmission spectra of IR reflector with PVA–PVK alignment layer under extra UV irradiation with different UV dose.
